# The Substituting Druggist

**Published:** 1903-06

**Authors:** 


### EDITORIAL NOTES AND COMMENTS.

The Business office of THE JOURNAL-RECORD is No. 64 Marietta Street.

The Editorial office is 318-319-320 The Prudential.

Address all Business communications to Dr. M. B. Hutchins, Mgr.

Make remittances payable to THE ATLANTA JOURNAL-RECORD OF MEDICINE.

On matters pertaining to the Editorial and Original Communications address Dr Bernard Wolff, Atlanta.

Reprints of original articles will be furnished at cost price. Requests for the same should always be made *on the manuscript*.

We will present, post-paid, on request, to each contributor of an original article, twenty (20) marked copies of THE JOURNAL-RECORD containing such article.

### THE SUBSTITUTING DRUGGIST.

THE increasing urgency that physicians do their own dispensing is forced upon our minds by the existence, we fear in the majority, of the kind of druggist mentioned in the title ot this editorial. The complaints of substitution by druggists are not limited to the manufacturers of proprietary medicines, as has been so often charged. The physician, whose friend the druggist pretends to be, suffers in his failure to get desired and expected results because the remedies he prescribes are not dispensed. In his acts of substitution the druggist, in his selfish greed, constitutes himself an enemy of the physician and unworthy of patronage or consideration. Having no authority under the laws to treat the sick, the druggist has no right under any circumstances to alter a physician's prescription, usurping the functions of the latter. As a humane being, it is his duty to notify the physician of any error in a prescription, fill the correct prescription, and leave the responsibility where it belongs and should belong, with the legalized practitioner.

There is, further, a criminal side to this question. The druggist who substitutes inert or inferior drugs for those prescribed not only commits a dishonest, even larcenous act; he tampers with human life, and may even be guilty of voluntary manslaughter.

Of course, there are honest and conscientious druggists who can be relied upon to fill a prescription as directed, and these have and deserve the highest esteem. They, however, share the opprobrium brought upon their profession by the substituting druggist.

It is not pleasant to feel, when giving a prescription to a patient, that the success desired depends upon the character of the druggist who will fill the prescription. Dispensing for himself is a difficult, almost impossible thing for the busy physician, yet, given honest drugs, his results would justify this measure. One other recourse would be to prescribe only original packages or bottles of proprietaries, to insist upon "C. P." drugs to be added, have tests made occasionally, and eternally blacklist every druggist proven guilty of substitution.

No druggist has the right to arrogate to himself the judgment that another drug or preparation is "just as good" as the one ordered. Your coal dealer would have no justification in supplying you, at $6.00 a ton, slaty, inferior coal, worth $3.00, at the first-named price when you had ordered the best. If you order pure water and the dealer delivers infected, possibly sewerage water, he would be none the more criminal, rather less, than the man who would furnish inferior drugs to the sick. If the old joke of the doctors killing people is to be perennial, let them have the responsibility unmixed with gratuitous help from the druggists.

